# Head Up Cardiopulmonary Resuscitation: Novel Physiological Discoveries and Latest Clinical Outcomes

**DOI:** 10.18103/mra.v13i8.6907

**Published:** 2025-08

**Authors:** Pouria Pourzand, Anja Metzger, Keith Lurie

**Affiliations:** 1 Department of Emergency Medicine, Lehigh Valley Health Network, Bethlehem, PA; 2 Department of Emergency Medicine, University of Minnesota, Minneapolis, MN

## Abstract

Despite decades of research, survival rates following cardiac arrest remain dismal. The recent use of pressure-volume (PV) loop analysis has provided important insights into the underlying physiology of cardiopulmonary resuscitation (CPR). During conventional CPR (C-CPR), ventricular ejection is limited, contributing to poor circulatory effectiveness. In contrast, automated head-up CPR (AHUP-CPR)—which combines active compression-decompression (ACD) CPR, an impedance threshold device (ITD), and controlled head-thorax elevation—generates favorable negative intrathoracic pressure, promoting cerebral venous drainage and increases right heart preload, resulting in enhanced stroke volume, cardiac output, and end-tidal CO_2_ (ETCO_2_) by improving ventricular–pulmonary–arterial circulation. This article summarizes recent advances related to AHUP-CPR with a focus on novel physiological findings derived from intraventricular conductance catheter recordings during ventricular fibrillation, C-CPR, and AHUP-CPR. The PV loop observations shed new light on the mechanisms through which AHUP-CPR improves circulatory efficiency and neurological outcomes.

## Introduction:

Survival after cardiac arrest is highly dependent upon the no-flow time and the amount of blood flow delivered to the heart and brain once CPR is initiated ^1–3^. Even when the time from collapse and the call for help is rapid, the amount of blood flow delivered to the vital organs during traditional CPR, including the heart and brain, remains too low for a successful long-term outcome ^4–7^. Results from currently used conventional CPR (C-CPR) techniques, whether manual or mechanical, have remained essentially unchanged for 60 years ^7,8^. Manual C-CPR relies on performing a physically demanding and challenging method: it is necessary but generally insufficient by itself to provide sufficient blood flow to restore full life ^4,5,9–12^.

**Figure. 1:**
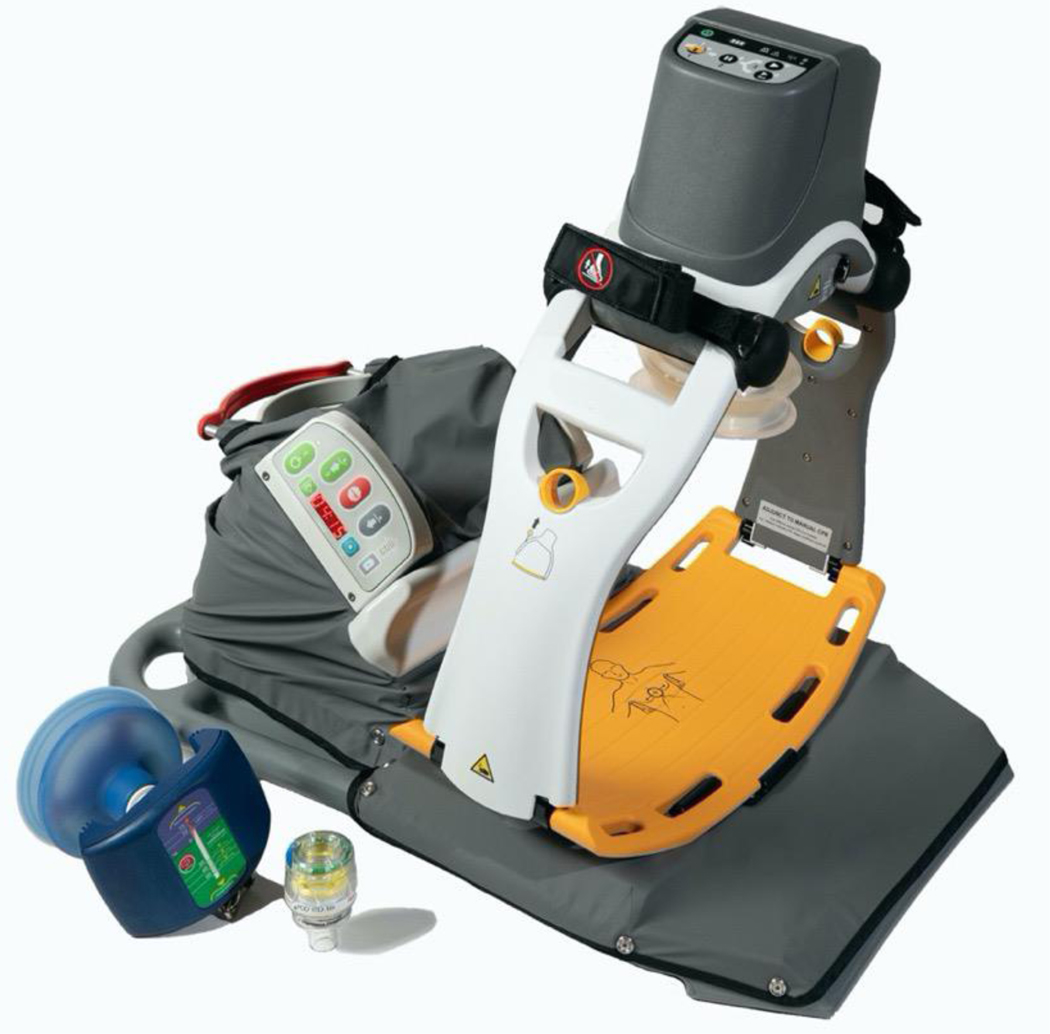
Automated head-up CPR patient positioning system, the EleGARD, coupled with automated CPR. The manual automated compression-decompression (ACD) CPR device and the impedance threshold device (ITD) are also shown on the left.

Automated head-up CPR (AHUP-CPR), delivered with a device that automatically elevates and properly positions the head and thorax, a suction cup-based active compression decompression (ACD) CPR device, and an impedance threshold device (ITD), was created and introduced as a more effective complementary approach to C-CPR that significantly enhances blood circulation to the heart and brain while simultaneously lowering intracranial pressures, thereby markedly improving the likelihood of survival with favorable neurological function after cardiac arrest ^13–22^ (Figure 1).

This review is focused on some recent clinical outcomes with AHUP-CPR but primarily on some newly discovered mechanisms that underlie the clinical benefits of AHUPCPR. These new physiological observations related to the cardiovascular effects of AHUP-CPR are based upon use of intraventricular conductance catheters to measure intraventricular pressures and volumes, and indirectly ventricular compliance and the coupling between the cardiac ventricles and the vasculature

## Background:

In 2014, the merits of an engineering concept for a new Korean stretcher were assessed, which could be folded and bent the legs upwards at 90 °, intended to address the challenge of performing CPR in the small elevators in high-rise apartments in Seoul, Korea. The resultant debate regarding ‘feet up’ versus ‘head up’ led to the first pig studies on the position of the body during CPR ^23^.

**Figure 2:**
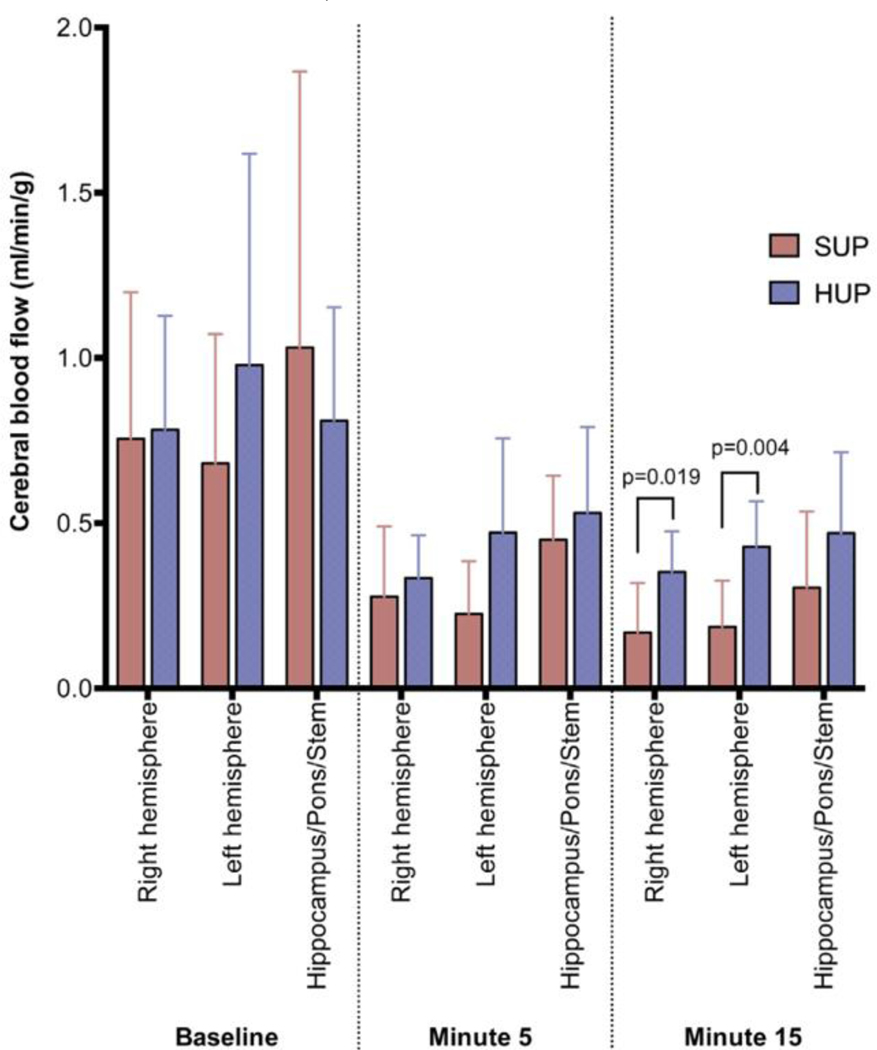
The blood flow to various areas of the brain with automated head-up CPR (HUP) vs automated compression-decompression CPR with impedance threshold device in supine and flat position (SUP) during a prolonged CPR effort (Moore et al. Resuscitation. 2017; 121:195–200)

A profound increase in ICP and a decrease in CerPP were observed with the ‘feet up’ position during CPR ^23^. In contrast, the opposite was observed in a whole body 30° ‘head up’ tilt position, where there was a marked decrease in ICP and an increase in CerPP and cerebral blood flow versus flat and ‘feet up’ positions ^23^. Subsequent animal studies showed that the combination of ACD-CPR plus gradual elevation of the head and thorax in a controlled manner, plus an ITD, doubled blood flow to the brain compared with ACD+ITD CPR performed in the flat position ^16–18,24,25^ (Figure 2). In addition, neurologically favorable survival rates were 6-fold higher with the new concept of AHUP-CPR versus C-CPR in the flat position ^25^. Importantly, recent studies have shown that AHUP-CPR needs to be provided as soon as possible by basic life support providers. In one pig study comparing early AHUP-CPR to mimic first responder deployment of this new approach versus starting high-quality C-CPR right away and delaying AHUP-CPR to mimic starting the new approach by Advanced Life Support providers, the hemodynamics and 24-hour survival rates which markedly better when AHUP-CPR was provided as soon as possible ^20^.

There are multiple putative mechanisms of action of AHUP-CPR. These include: 1) enhancement of venous blood from the brain to the thorax which lowers intracranial pressure (ICP), enhances cerebral blood flow and protects the brain, which is why head elevation is used in patients with traumatic brain injury 2) increased cardiac preload and better blood flow through the cardiopulmonary circuit, which is why patients in heart failure like to sit up, 3) better ventilation as the diaphragm moves downward creating more lung volume, less atelectasis, and lower airway opening pressures, together making ventilation more effective and reducing VQ mismatch, 4) reduction in right-and left-side high venous and arterial pressure waves with each compression, thereby lowering the chances for a ‘concussion with every compression’, and 5) greater cardiac output resulting in better myocardial perfusion and a higher likelihood of sustain return of spontaneous circulation ^4,13–16,23,26–33^.

### Recent Clinical Data:

Multiple positive clinical outcomes studies with this device combination have been published ^19,21,22,34^. In 2019, the ELEGARD was cleared for use by the United States Food and Drug Administration and thereafter introduced to multiple EMS systems. Outcomes have been tracked with an IRB-approved AHUP-CPR Registry. The outcomes have been analyzed from >1450 patients treated with AHUP-CPR with a focus on time to AHUP-CPR device deployment time ^19,35^. With data from 12 US EMS systems in 8 states where 911 call to AHUP-CPR device placement times were routinely tracked, we observed that shorter times to AHUP-CPR device placement were associated with higher return of spontaneous circulation, hospital discharge rates, and neurologically-favorable survival rates ^19^. Like time to defibrillation for a shockable rhythm, time to AHUP-CPR is a critical and independent determinant of a successful outcome. Unlike an automated external defibrillator, AHUP-CPR works for all heart rhythms. The combined clinical data demonstrate a consistently strong and positive association between early AHUP-CPR delivery by first responders and neurologically-favorable survival benefit, regardless of the first recorded rhythm ^19,21^.

**Figure 3:**
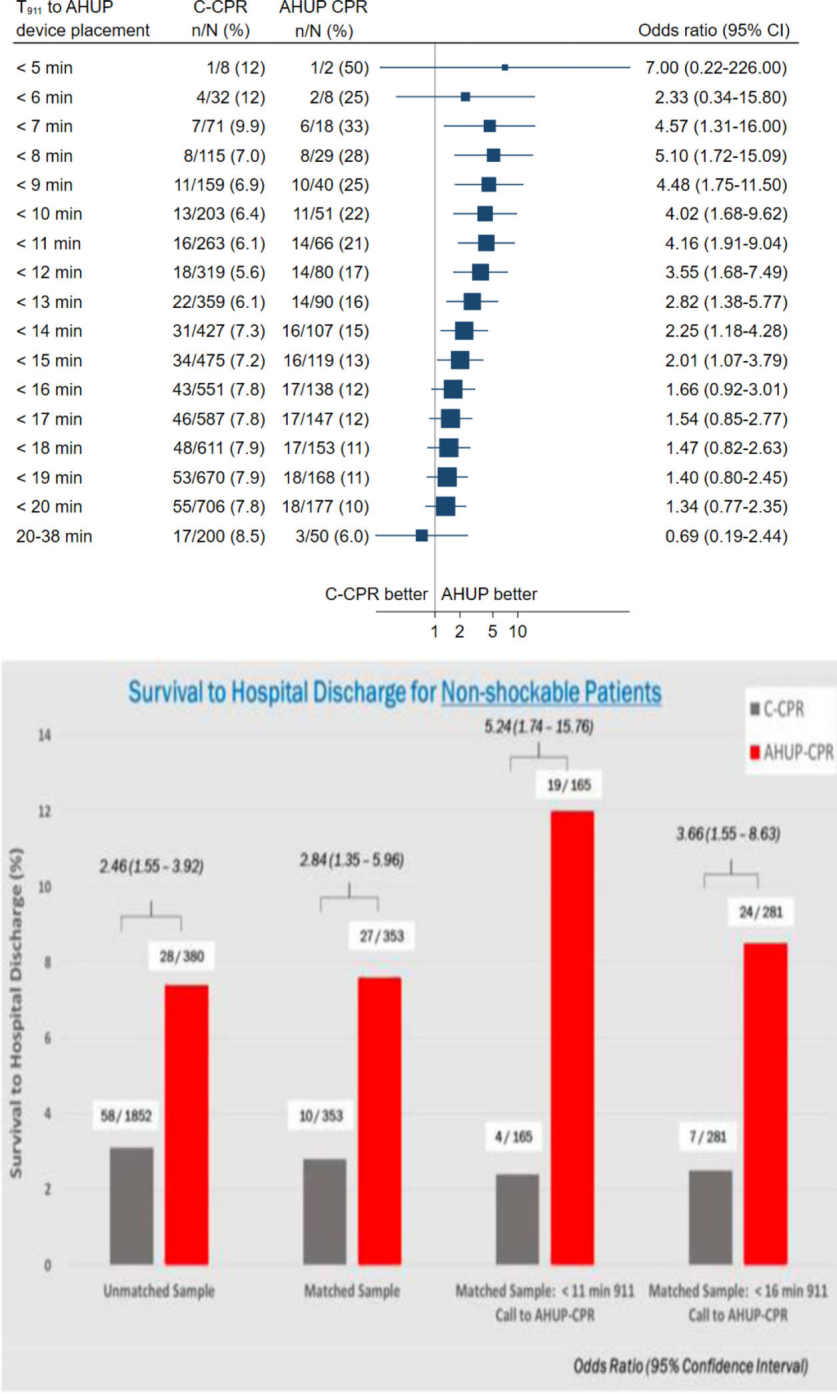
Survival with neurological favorable function divided based on time of 911 call to automated head-up CPR bundle placement (top; Moore et al. Resuscitation. 2022; 179–9–17). Survival to hospital discharge for non-shockable rhythms with conventional CPR vs automated head-up CPR (bottom; Bachista et al. Critical Care Medicine. 2024; 52(2): 170–181).

As shown in Figure 3, from one of the first published clinical observation trials, the likelihood of favorable neurological outcomes regardless of the presenting rhythm is >4 times higher with AHUP-CPR when it is initiated in <11 minutes from the 9–1-1 call. Given the average time from a 9–1-1 call to first responder CPR is ≤8 min in many cities, the potential for increasing survival rates with AHUP-CPR is enormous. Another observational study with 380 cardiac arrest patients that presented with a non-shockable first recorded rhythm also demonstrated a strong association between early AHUP-CPR use and survival, as shown in Figure 3 ^22^. Of note, patients in that study with an initial rhythm of pulseless electrical activity, about 25% of all OHCA, had a 10% neurologically favorable survival rate with AHUP-CPR versus ~3% with C-CPR.

### Recent Mechanistic Insights into AHUP-CPR using Pressure-Volume (PV) loops:

To assess cardiovascular function during CPR, researchers have traditionally used systemic blood pressure and blood flow ^19,36,37^. While these metrics are valuable, they offer only a partial view of cardiovascular performance. Specifically, there are several methods in the literature to measure generated ventricular pressure, volume and/or cardiac output (CO), such as Doppler ultrasound, microspheres, and thermodilution ^38–43^. However, these methods incorporate several limitations in accurately measuring ventricular pressure, volume and other hemodynamic indices, such as compliance, contractility efficiency and/or relationship between ventricles and arterial circulation.

**Figure 4:**
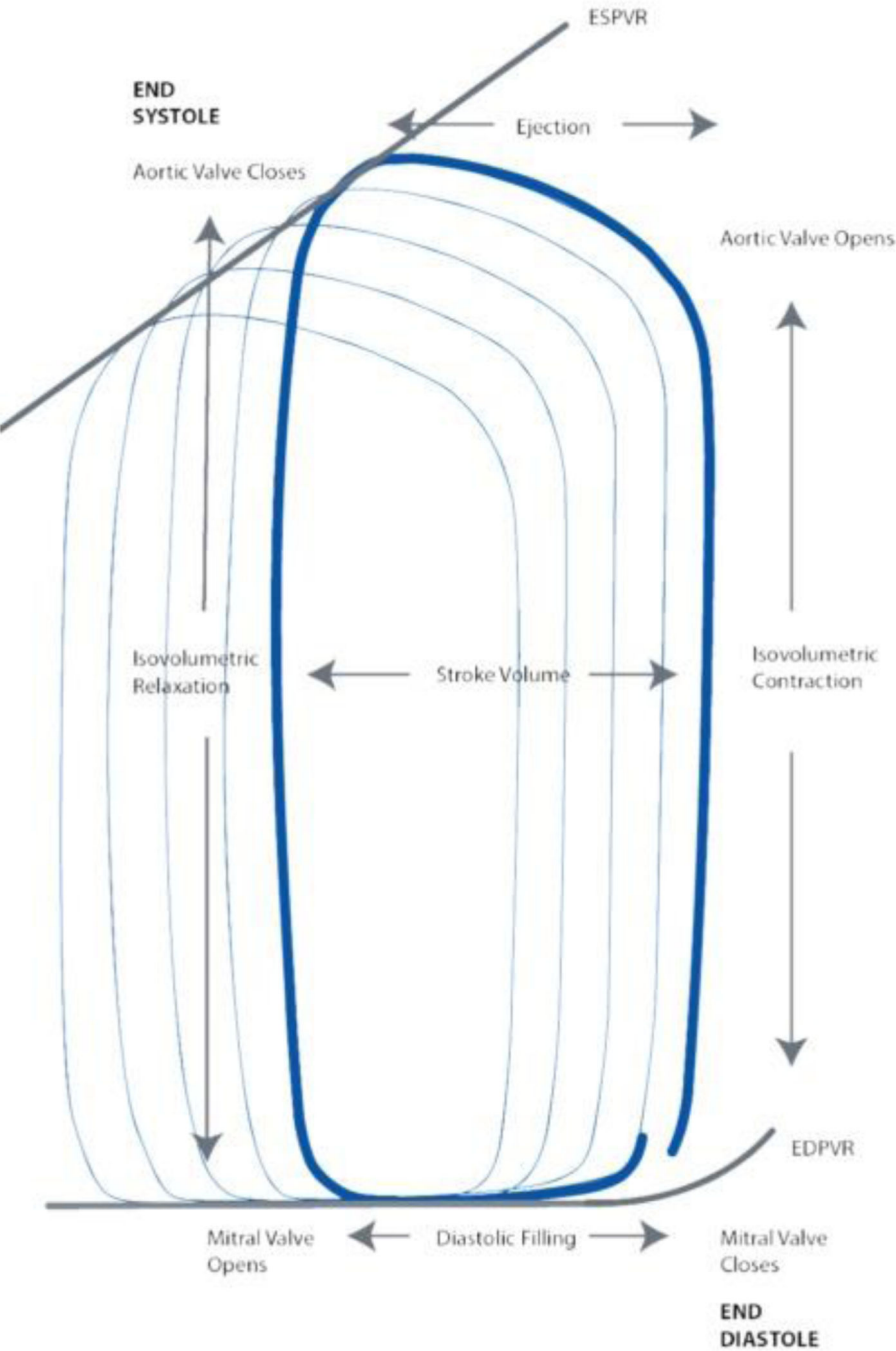
A schematic of pressure-volume loop under normal physiologic conditions (CD Leycom, Hengelo, Netherlands).

Pressure-Volume (PV) loops (Figure 4) can provide a more comprehensive assessment of the mechanisms at work during CPR by capturing hemodynamic parameters that are difficult to measure through other techniques as some consider PV loop recordings using intraventricular conductance catheters as the “gold standard” for quantitative measurement of intracardiac hemodynamics during normal sinus rhythm ^44,45^. A key advantage of PV loops is their ability to provide precise instantaneous quantitative data rather than just qualitative observations. As a result, they are considered one of the most comprehensive tools for evaluating cardiac function and overall hemodynamics. Moreover, the accuracy of measuring ventricular volumes and pressures through PV Loops with a conductance catheter has been corroborated by existing research during normal sinus rhythm ^41,44–47^.

### PV Loop Methodology:

**Figure 5:**
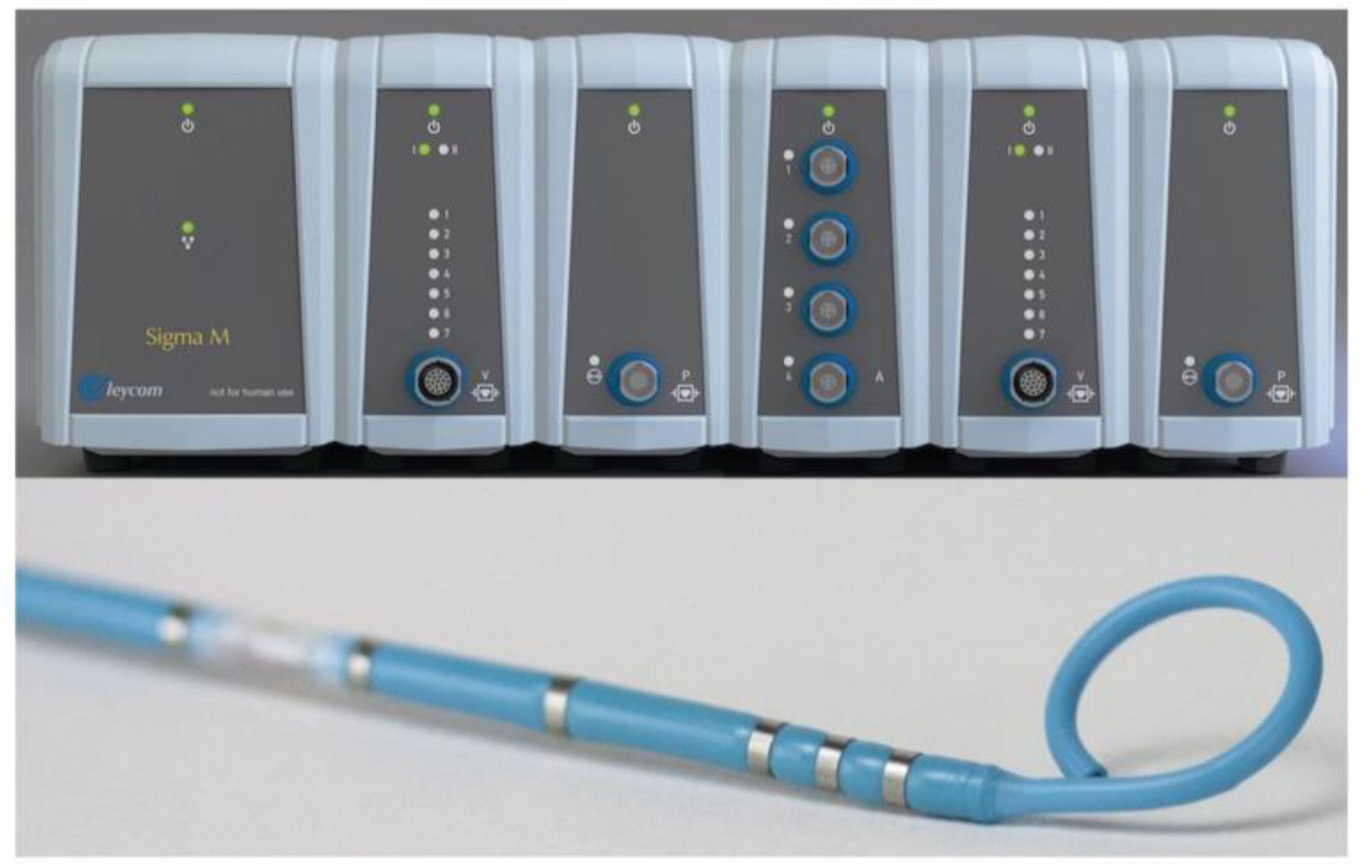

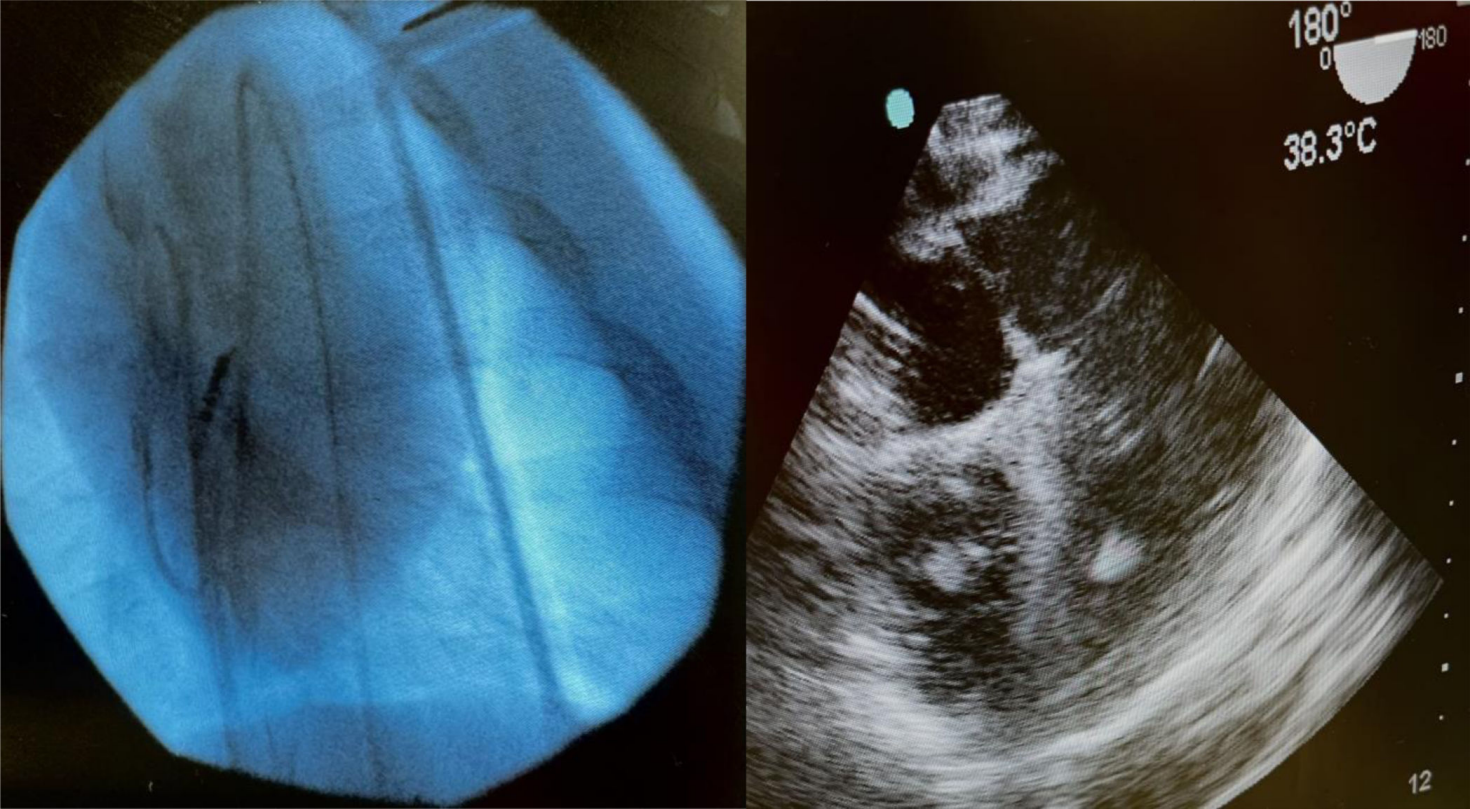
Biventricular pressure-volume loop system along with conductance catheters (top, CD Leycom, Hengelo, Netherlands), placement confirmation under fluoroscopy (bottom left) and transesophageal echocardiography (bottom right).

We recently introduced the concept of using a high-fidelity intraventricular PV loop system during CPR for the first time to measure instantaneous pressure and volume generated inside the right and left ventricles in conjunction with comprehensive peripheral hemodynamic monitoring ^48,49^. In those studies, biventricular pressure-volume (PV) loops were obtained using 7Fr conductance catheters in the right and left ventricles (CA-71083-PL; CD Leycom, Hengelo, The Netherlands) equipped with 12 electrodes and an integrated high-fidelity pressure sensor, connected to the Inca^®^ signal processing unit (CD Leycom, Hengelo, The Netherlands). This approach can be used to determine both ventricular volumes and pressures on a millisecond basis during each compression-decompression cycle (Figure 5).

### PV Loop Anatomy – Normal Physiological Conditions:

Under normal physiological conditions in the spontaneously beating heart, the intraventricular cycle, illustrated by the PV loop (Figure 4), begins with the opening of the mitral and tricuspid valves during diastole, allowing blood to flow into the ventricles. Ventricular filling continues until the closure of the mitral and tricuspid valves, marking the onset of isovolumic contraction. During this phase, intraventricular pressure rises sharply until it surpasses the threshold required to open the aortic and pulmonary valves, leading to ventricular ejection of blood into the systemic and pulmonary circulation. At the end of systole, as ventricular pressure drops, the aortic and pulmonary valves close, initiating isovolumic relaxation. This phase prepares the ventricles for the next cycle by reducing pressure before the reopening of the atrioventricular (AV) valves for the next diastolic filling phase ^50^.

### PV Loop Anatomy – During Cardiac Arrest and CPR:

During cardiac arrest and ventricular fibrillation (VF), the physiology in the absence and presence of CPR is markedly different. The ventricles undergo several changes during VF. For example, RV size and volume increase likely due to venous return and continued auricular contractions, especially during the first few minutes ^51,52^. Studies have reported inconsistent findings for LV size and volume, pointing toward less size and volume changes, perhaps limited by anatomy, chamber compliance, myocardial thickening, prominent RV dilation, as well as intraventricular pressure dynamics ^53^. Our findings were similar, showing that ventricular volumes increased initially, peaked at about 2 minutes, and then decreased thereafter.

**Figure 6:**
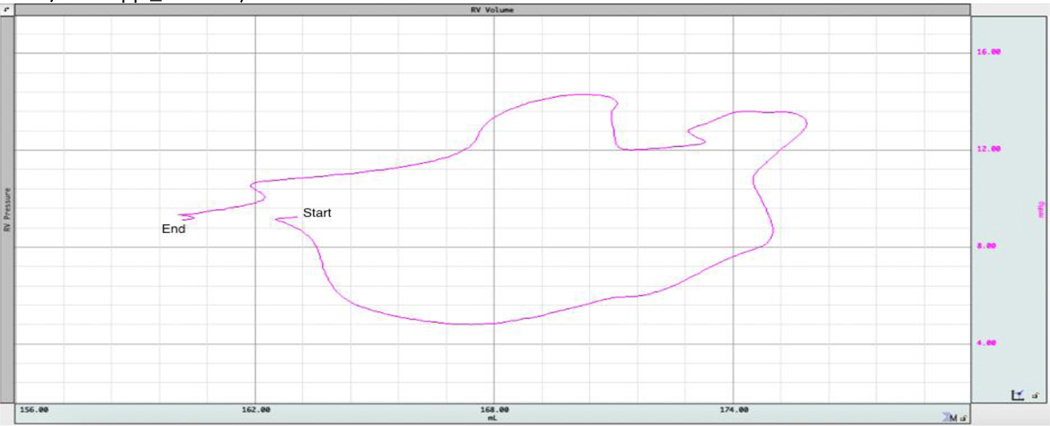

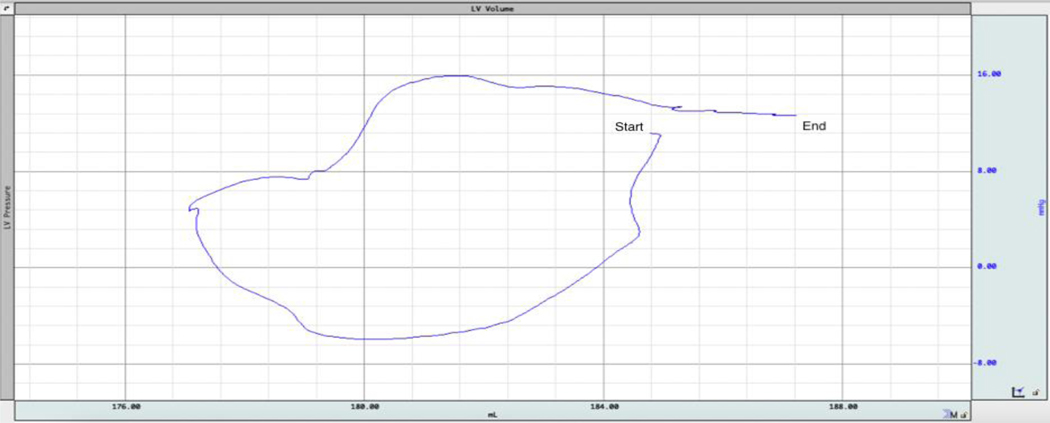
Gasping effect on right (top) and left (bottom) ventricles pressure-volume loops (Pourzand et al. Circulation. 2024; 150.suppl_1.Sa201)

We recently explored and demonstrated how gasping adds to this complexity ^54^. Agonal respiration or gasping often begins within 1–2 minutes of untreated cardiac arrest ^55^. Gasping lowers intrathoracic pressure, especially in the presence of inspiratory resistance with an ITD, which increases right ventricular volumes. On the other hand, LV volume decreased initially during the negative pressure phase of the gasp and increased towards the end of gasping (Figure 6). This initial decrease in LV volume may be partly due to RV overdistension and/or retrograde flow ^53^.

### PV Loop Anatomy – Conventional CPR

We found that, during CPR, the normal pressure-volume dynamics are profoundly altered, primarily because the heart no longer generates its own pressure gradient, and blood flow relies entirely on external chest compression and decompression ^9,56^. CPR lacks true isovolumetric phases due to continuous pressure changes from external compressions. Since ventricular contraction is passive, intraventricular pressure does not rise intrinsically; rather, it fluctuates in response to thoracic compressions and recoil. We also found that PV loops’ characteristics and shape are significantly varied based on the CPR method.

**Figure 7:**
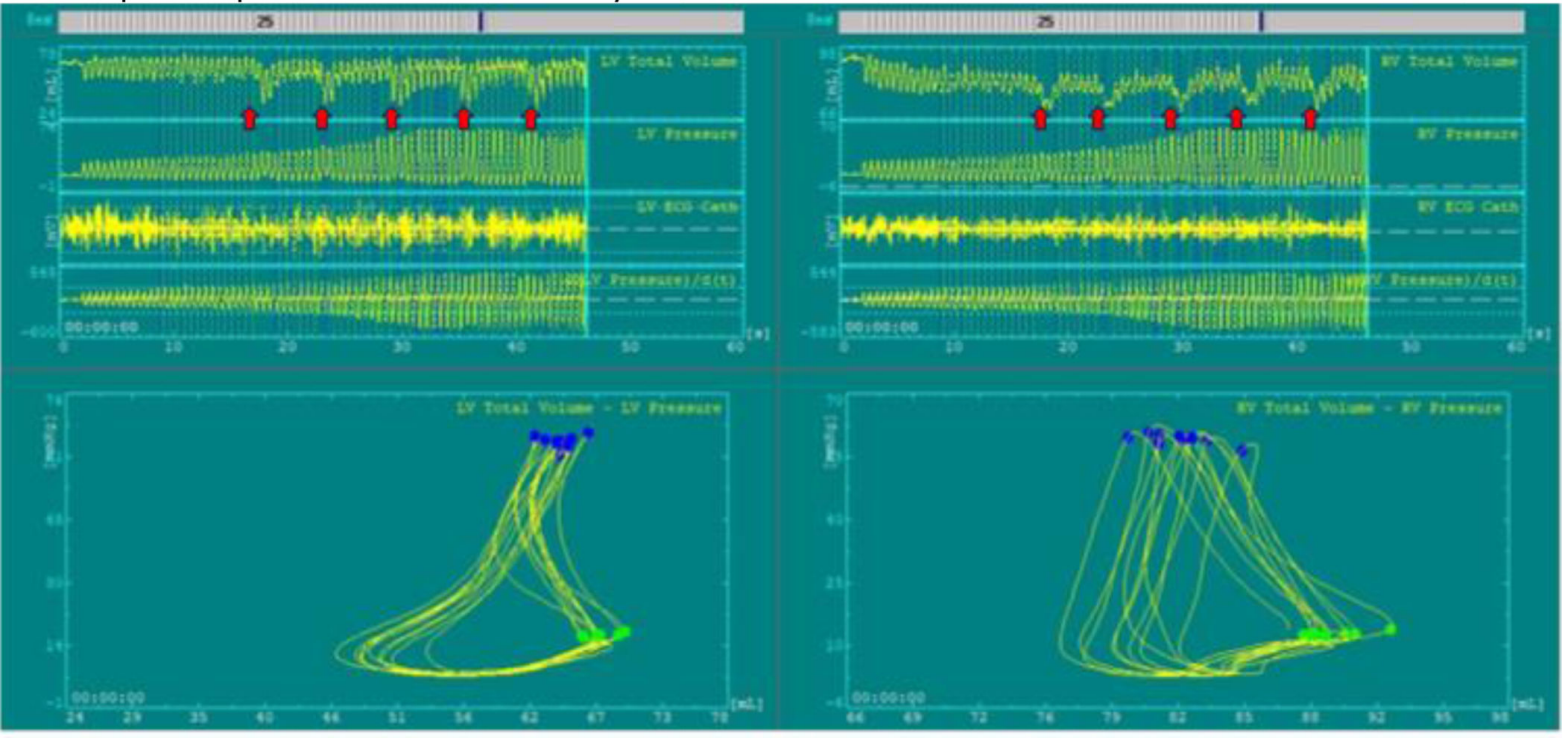
Left (LV) and right (RV) ventricular pressure-volume loops and channels at the beginning of CPR. Markers indicate positive pressure ventilation delivery and it’s effect of intraventricular volume

At the start of C-CPR in our studies using a pig model of cardiac arrest, the volume inside the ventricles decreases initially with each compression and there is simultaneously limited blood return to the ventricles during decompression (Figure 7). This imbalance persists until a new, low-flow hemodynamic equilibrium is achieved. The arterial vasculature (i.e., pulmonary artery and aorta) is not very compliant and limits the transmission of forward flow during compressions and contributes to ineffective CO generated by manual or conventional CPR.

These mechanical limitations result in a significantly impaired cardiovascular function during C-CPR. As a result, SV and overall CO are reduced to about 40% of normal values during sinus rhythm. Left ventricular systolic pressure is substantially reduced (~50–60 mmHg), while right ventricular systolic pressure is paradoxically elevated (~60–70 mmHg), which may potentially reflect proximity to the compressor as well as increased pulmonary resistance. Furthermore, as isovolumetric phases are poorly defined due to continuous pressure fluctuations, pressure rises sharply with a steep volume drop. Both end-compression and end-decompression volumes are elevated, indicating incomplete ventricular ejection and impaired venous return, likely due to retrograde flow. Pressure–volume relationships at both end-systole and end-diastole are suboptimal, pointing to reduced contractility and compliance. In addition, arterial elastance (Ea) is elevated and end-systolic elastance (Ees) is diminished, leading to poor ventriculo-arterial (VA) coupling and inefficient ventricular-vascular interaction ^49^. These derangements manifest clearly in PV loops, such that PV loops during C-CPR are reduced in size and vertically oriented, indicating diminished SV and altered filling dynamics. Lastly, our findings also showed that ventricular volume and pressures vary greatly with each positive pressure ventilation (Figure 7).

Of note, our CPR protocol starts with a compression depth of about 3.5 cm and a compression rate of 100/min and to minimize rib fractures the depth is increased to ~5.0 cm over 60 seconds, at which time ventricular volumes are at an equilibrium, with about 25% less volume than observed at the start of CPR.

### PV Loop Anatomy – AHUP-CPR

**Figure 8:**
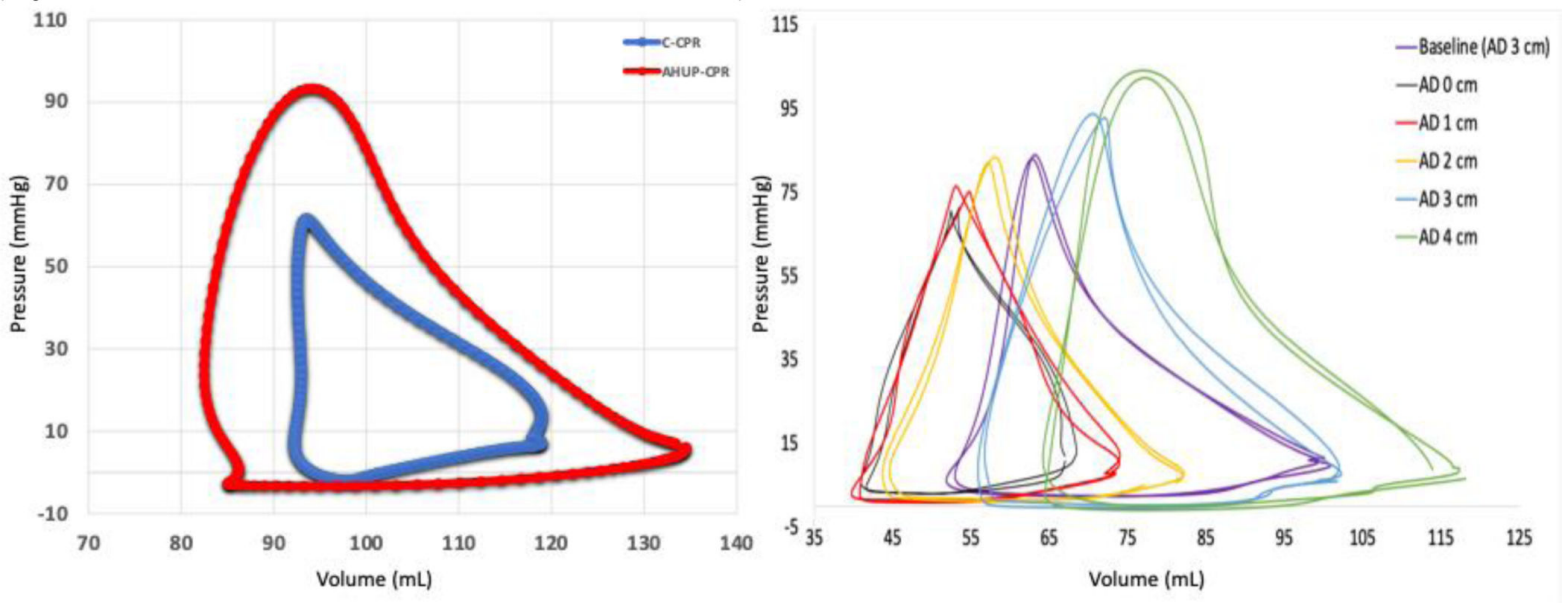
A comparison between pressure-volume loops generated in the right ventricle by conventional CPR vs automated head-up CPR (left). Effect of different levels of active decompression (AD) on pressure-volume loops in the right ventricle (right; Pourzand et al. Resuscitation. 2024; 110–3-24).

Compared to C-CPR, AHUP-CPR resulted in higher SV/CO, leading to lower ECompV while enhancing and preserving EDecompV proportional to the degree of SV enhancements. Compression efficiency improves as reflected by higher End-Compression Pressure (ECompP) and End-Compression Pressure-Volume Relationship (ECompPVR). Simultaneously, ventricular distensibility/compliance increased, demonstrated by lower EDecomP and EDecompPVR. VA coupling also significantly improved with lower Ea/Ees as shown in Figure 8.

Furthermore, we found that the addition of each 1 cm of active decompression (AD) to ITD enhances right and left ventricular preload, compliance, contractility efficiency, SV/CO and VA coupling incrementally, with peaked hemodynamics achieved with 4 cm of AD (Figure 8). With full active decompression during AHUP-CPR, we observed a lower EDecompPVR, which serves as an index of ventricular compliance. EDecompPVR was also associated with higher volumes inside ventricles, highlighting enhanced compliance and volume at the end of decompression.

Correlative analysis further supported these findings. Our findings showed a significant positive correlation between ECompP and ECompPVR in both ventricles and ETCO2, as well as CerPP, which was correlated with lower volume inside the ventricles. ECompPVR was also associated with VA coupling, which suggests that higher contractility efficiency leads to better VA coupling, indicating that greater compression efficiency supports more effective ventriculo-vascular interaction.

VA coupling, which characterizes the interaction between the cardiac contractility and the arterial circulation afterload, reflects cardiovascular efficiency ^57^. In our study, C-CPR resulted in a significant VA decoupling with C-CPR, which was reversed by AHUP-CPR. Full ACD CPR during AHUP-CPR further improved VA coupling incrementally, with optimal results achieved with 4cm of AD. The improvements in VA coupling were associated with higher SV in both ventricles, as well as higher ETCO2, particularly in the RV. Additionally, improved VA coupling was also associated with higher aortic compression and decompression pressures, and consequently coronary and cerebral perfusion pressures, highlighting enhanced circulation to the lungs, heart and brain. Together, these findings underscore the central role of VA coupling in optimizing circulation to the lungs, heart, and brain during AHUP-CPR ^48,49^.

Recognizing the terminology can get confusing, we have tried in Table 1 to compare PV loop findings during normal sinus rhythm with those we observed during C-CPR and the AHUP-CPR.

These results we observed during the PV loop studies were consistent with the putative mechanisms associated with the physiology of cardiac arrest, C-CPR, and AHUP-CPR ^4,16–18,20,38,39,56^. After 10 minutes of untreated VF, blood accumulation in the heart and C-CPR is insufficient to pump the blood out of the ventricles. The utilization of ACD+ITD generates a favorable negative intrathoracic pressure and increased preload based upon drainage of venous blood from the brain, thereby enhancing blood flow to the right heart and pushing more blood out of both ventricles, which is highlighted by an increase in the SV/CO and ETCO2 during AHUP CPR. The effects of AHUP-CPR are due in part to gravity, which draws more blood towards the heart, as evident by lower atrial and ventricular pressures during the decompression phase of AHUP-CPR, along with higher EDV, and therefore increasing the output. The physics of AHUP-CPR favors greater VA coupling and the efficiency of the overall pumping system and delivery of oxygenated blood to the heart and brain. Further research is needed to better understand the mechanism of CPR through the lens of the PV loop studies.

## Conclusion:

The recent use of PV loops has helped to further elucidate the physiology of gasping during untreated VF, C-CPR, ACD+ITD CPR in the flat position, and AHUP-CPR. The data generated with the PV loops supports the hypothesis that during cardiac arrest, early use of AHUP-CPR can restore hemodynamics, blood flow, VA coupling, and cardiac contractility and relaxation to levels close to those observed during normal physiological states, in the absence of cardiac arrest. Based upon these collective observations, further refinement of the non-invasive suite of tools needed for AHUP-CPR (e.g. lighter weight, easier to deploy, more compact, more affordable) offer great promise to even better clinical outcomes after cardiac arrest in the future.

## Figures and Tables

**Table 1:** Summary of pressure-volume loop parameters comparing conventional CPR (C-CPR) vs automated head-up CPR (AHUP-CPR).

Parameter	C-CPR	AHUP-CPR
**Loop shape**	Smaller, vertical loops	Larger than C-CPR, more optimized loops
**Stroke Volume Cardiac Output**	~40% of normal	Higher than C-CPR (improves with AD), about to 80% (LV) and 90% (RV) of normal
**End Systolic/Compression Pressure**	LV~50–60 mmHg RV ~60–70 mmHg	Higher than C-CPR and better matched with afterload
**End Diastolic/Decompression Pressure (EDecompP)**	Elevated	↓; reflects better compliance
**Isovolumetric Phases**	Absent	Absent
**End Systolic/Compression Volume (ECompV)**	↑ (incomplete emptying)	↓ with higher SV and better compression efficiency
**End Diastolic/Decompression Volume (EDecompV)**	↑ compared to baseline (poor emptying/retrograde flow)	Maintained or ↑ with improved preload/compliance
**End Systolic/Compression Pressure-Volume Relationship (ECompPVR)**	Suboptimal	↑; positively correlates with CO and perfusion
**End Diastolic/Decompression Pressure-Volume Relationship (EDecompPVR)**	High (low compliance)	↓; more compliant ventricles with higher volumes (↑ with AD)
**Contractility Efficiency**	Low	Improved; enhanced ECompPVR and SV
**VA Coupling (Ea/Ees Ratio)**	Poor	Improved; optimal with 4 cm AD

## Abbreviations and terminology used:

ACD: automated compression-decompression
AD: active decompression
AHUP-CPR: automated head-up CPR
C-CCPR: conventional CPR
CO: cardiac output
Ea: arterial elastance
ECompP: end-compression pressure
ECompPVR: end-compression pressure-volume relationship
ECompV: end-compression volume
EDecompP: end-decompression pressure
EDecompPVR: end-decompression pressure-volume relationship
EDecompV: end-decompression volume
Ees: end-systolic elastance
ETCO2: end-tidal CO2
ITD: impedance threshold device
PV: pressure-volume
SV: stroke volume
VA: ventriculo-arterial
VF: ventricular fibrillation
